# Mining the Public Mind: A Text-Mining Approach to Dental Implants and Dentures

**DOI:** 10.3390/dj14060352

**Published:** 2026-06-09

**Authors:** Hyun-Jun Kong

**Affiliations:** Department of Prosthodontics and Wonkwang Dental Research Institute, School of Dentistry, Wonkwang University, Iksan 54538, Republic of Korea; zsfvzsfv@naver.com; Tel.: +82-0638505938

**Keywords:** data mining, medical informatics, sentiment analysis, dental implants, dentures

## Abstract

**Background/Objectives**: This study aimed to comparatively analyze online information regarding dental implants and dentures utilizing text-mining techniques. **Methods**: An automated text-mining program was employed to collect and process data using the Korean keywords for “implant” and “denture.” Data sources included major search engines, social networking services, and YouTube (Google LLC, Mountain View, CA, USA). A total of 9941 data points for dental implants and 9783 for dentures were retrieved. The analytical approach included word cloud generation, term frequency-inverse document frequency (TF-IDF) analysis, semantic network analysis, and sentiment analysis. **Results**: For implants, “dental clinic,” “treatment,” “surgery,” and “insurance” emerged as highly relevant keywords. In contrast, queries regarding dentures frequently included the term “implant,” alongside top-ranking, age-related terms such as “abnormality” and “discomfort.” TF-IDF analysis revealed that “surgery” and “procedure” ranked higher for implants, whereas “insurance” ranked higher for dentures. Sentiment analysis indicated a predominantly positive public perception of implants (63.09% positive, 36.91% negative), whereas dentures elicited a largely negative sentiment (40.70% positive, 59.30% negative). **Conclusions**: The text-mining analysis revealed distinct public perceptions regarding the two treatments. Implants were primarily associated with surgical procedures and positive sentiments, whereas dentures were more closely linked to insurance considerations and negative experiences.

## 1. Introduction

According to the Glossary of Prosthodontic Terms, edentulism is defined as “the state of being edentulous; without natural teeth,” typically resulting from a multifactorial process involving biologic factors and trauma [1]. Edentulous patients often face significant challenges, including impaired mastication, altered phonetics, and decreased social interaction, alongside anatomical consequences such as residual ridge resorption and loss of facial support [2,3]. While conventional dentures have historically been the standard of care, their functional and aesthetic limitations become pronounced in patients with severe ridge resorption. Conversely, the advent and popularization of dental implants have driven a surge in demand, fueled by increasing patient awareness. For completely edentulous patients, implant-supported treatments offer distinct clinical advantages over conventional dentures, notably enhanced prosthesis stability, retention, and support, which translate to improved masticatory efficiency and overall patient convenience [4].

Conventional dentures offer a non-invasive, cost-effective approach for immediate rehabilitation; however, being tissue-supported, they often provide inferior masticatory efficiency and can cause discomfort or accelerate bone resorption. In contrast, dental implants are bone-anchored via osseointegration, preserving bone volume and delivering stability and masticatory function comparable to natural teeth. However, these functional benefits must be evaluated against inherent disadvantages such as the need for invasive surgery, prolonged treatment duration, and high financial costs.

Although the clinical literature consistently reports higher patient satisfaction with implant overdentures and implant-fixed prostheses compared to conventional dentures [5,6,7], there remains a paucity of research exploring how the general public—rather than existing patients or dental professionals—perceives these two treatment modalities. Investigating online data, which is readily accessible and spontaneously generated, presents a valuable avenue for understanding this broader public perception. When seeking medical information, individuals frequently turn to internet search engines for rapid and convenient answers.

With over three billion internet users globally, digital platforms have become a primary source of health information. This is particularly evident in South Korea, where an advanced information and communication technology (ICT) infrastructure and ubiquitous smartphone adoption have deeply integrated the internet into daily life. Consequently, the public heavily relies on search engines, social networking services (SNS), and platforms like YouTube (Google LLC, Mountain View, CA, USA) for medical insights [8,9,10]. In a previous study, we utilized Google Trends (Google LLC) to investigate public interest in dental implants; however, that approach was limited by Google’s relatively low search engine market share in South Korea and the platform’s restriction to analyzing only search volume trends rather than contextual meaning [11].

Text mining addresses these limitations by extracting statistically meaningful concepts, patterns, and high-quality relationships from unstructured data. Unlike standardized numeric data, unstructured data—such as text documents, images, and multimedia—requires advanced processing techniques. As a cornerstone of big data analysis and data science, text mining leverages methodologies like natural language processing (NLP), information retrieval, and machine learning to transform complex linguistic information into structured, analyzable data [12].

Therefore, the present study aimed to comprehensively analyze and compare public perceptions of dental implants and dentures in South Korea by applying text-mining techniques to data extracted from high-market-share search engines, SNS, and YouTube. The null hypothesis was that there would be no significant difference in the public’s perception of dental implants and dentures.

## 2. Methods

### 2.1. Data Collection and Refinement

This study was exempt from Institutional Review Board approval, as it exclusively utilized publicly available, anonymized data. Data collection and processing were conducted using Textom (version 5.0; IMC Inc., Daegu, Republic of Korea), an automated text-mining program. Leveraging NLP, this text-mining technology extracts valuable patterns and meaningful relationships from unstructured text data. The Korean equivalents of “implant” and “denture” were selected as the primary search keywords.

Data were retrieved retrospectively over a five-year period, spanning from January 2020 to December 2024. To capture a comprehensive cross-section of public discourse, data were sourced from major high-traffic online channels: Naver (Web, Blog, News, Café, and Knowledge Search; Naver Corp., Seongnam, Republic of Korea), Daum (Web, Blog, News, and Café; Kakao Corp., Jeju, Republic of Korea), Google (Web and News), Facebook (Meta, Menlo Park, CA, USA), X (formerly Twitter; X Corp., San Francisco, CA, USA), and YouTube.

**Figure 1:**
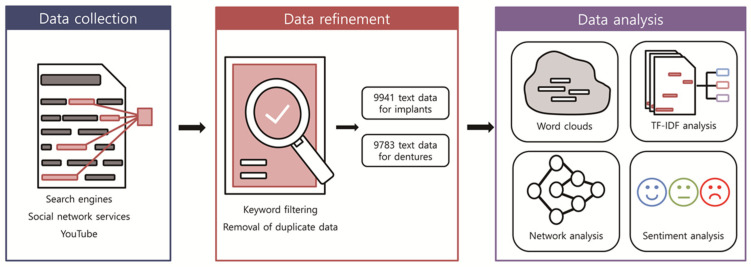
Overall scheme design of the text-mining workflow.

To ensure the integrity and relevance of the dataset, the raw text underwent a rigorous preprocessing phase. Initially, data filtering was applied to eliminate keywords completely unrelated to dental implants or dentures. Subsequently, data deduplication was performed; if multiple documents shared the same URL, all but a single unique instance were removed to prevent skewed results. This refinement process yielded approximately 10,000 valid textual data points for each keyword. Finally, morphological analysis was executed using MeCab-ko, an open-source Korean morphological analyzer adapted from its Japanese counterpart [13]. Regarding text-processing criteria, data extraction was strictly limited to nouns and adjectives, utilizing a custom stop-word dictionary to eliminate irrelevant terms and promotional boilerplate. Furthermore, to control for potential algorithmic and selection biases, we employed a multi-platform approach, collecting longitudinal data over five years combined with strict URL deduplication to ensure a balanced dataset. The overarching workflow encompassing data collection, preprocessing, and analysis is illustrated in Figure 1.

### 2.2. Data Analysis

#### 2.2.1. Word Clouds and Bar Chart Analysis

Word clouds are visual representations of text data wherein the size of each word corresponds to its frequency or relative importance within a dataset. As a straightforward and intuitive visualization tool, word clouds effectively distill large volumes of text, providing an immediate overview of the most prominent terms [14]. In this study, the extracted keywords were visualized using word clouds, supplemented by bar charts to quantitatively illustrate the precise frequency of these key terms.

#### 2.2.2. Term Frequency–Inverse Document Frequency (TF-IDF) Analysis

Term frequency–inverse document frequency (TF-IDF) is a statistical metric used to evaluate the relevance of a specific word to a document within a broader corpus. Widely utilized in information retrieval and text mining, TF-IDF facilitates keyword extraction, search result ranking, and document similarity identification. The metric is calculated by multiplying a word’s term frequency (TF) by its inverse document frequency (IDF) across the entire document set [15], expressed mathematically as:

TF-IDF = TF × ln(D/DF)

where TF represents the frequency of the word in a specific document, D is the total number of documents in the corpus, and DF denotes the number of documents containing the target word.

#### 2.2.3. Matrix Analysis and Semantic Network Analysis

Semantic network analysis visualizes the structural relationships and connective strengths between keywords by mapping them as interacting nodes. Following the calculation of keyword frequencies, matrix formulation and convergence of iterated correlations (CONCOR) analysis were executed using UCINET (version 6.0; Analytic Technologies, Harvard, MA, USA). Grounded in Pearson’s correlation coefficients, CONCOR analysis utilizes a co-occurrence matrix to uncover underlying network structures, cluster highly similar keyword groups, and evaluate the relational dynamics between these distinct clusters [16].

#### 2.2.4. Sentiment Analysis

Sentiment analysis leverages NLP, computational linguistics, and text analysis to systematically identify, quantify, and categorize subjective information and affective states within textual data [17]. In the present study, sentiment-based word-frequency analysis was conducted to evaluate the emotional tone of the extracted keywords. Sentiments were classified into two primary categories: positive and negative. The positive category comprised three dimensions (e.g., good feeling, interest, and joy), whereas the negative category encompassed six dimensions (e.g., sadness, disgust, pain, fear, anger, and fright). To accurately reflect emotional intensity, expressions corresponding to each sentiment were standardized using a weighted scoring system. For instance, within the “good feeling” dimension, a strongly expressive word such as “happy” was assigned a weight of 5 points, whereas a milder expression such as “so-so” received 1 point.

## 3. Results

**Figure 2:**
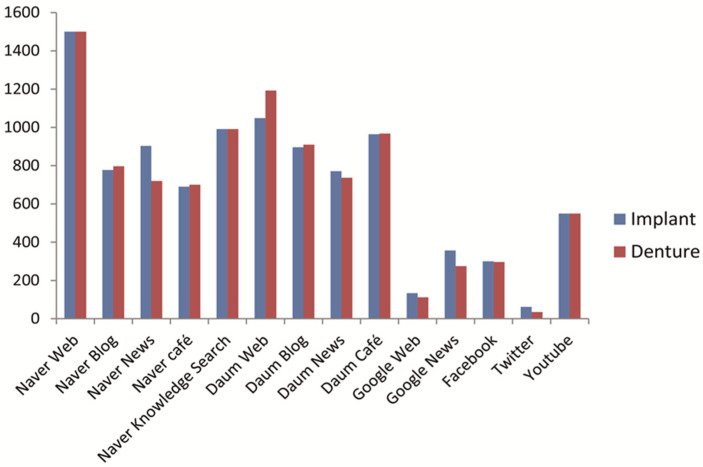
The number of datapoints collected for each channel used in this study.

Figure 2 illustrates the distribution of collected data points across the various online channels. A total of 9941 data points were retrieved for dental implants, alongside 9783 data points for dentures. For both treatment modalities, Naver emerged as the predominant source of information, accounting for approximately 50% of the total dataset.

### 3.1. Word Cloud and Bar Chart

**Figure 3:**
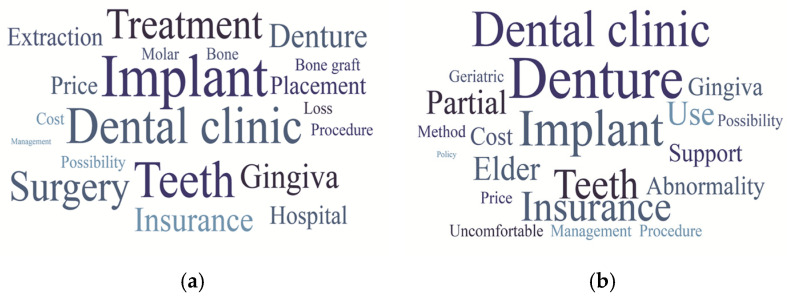
Word cloud images; (**a**) dental implants; (**b**) dentures.

**Figure 4:**
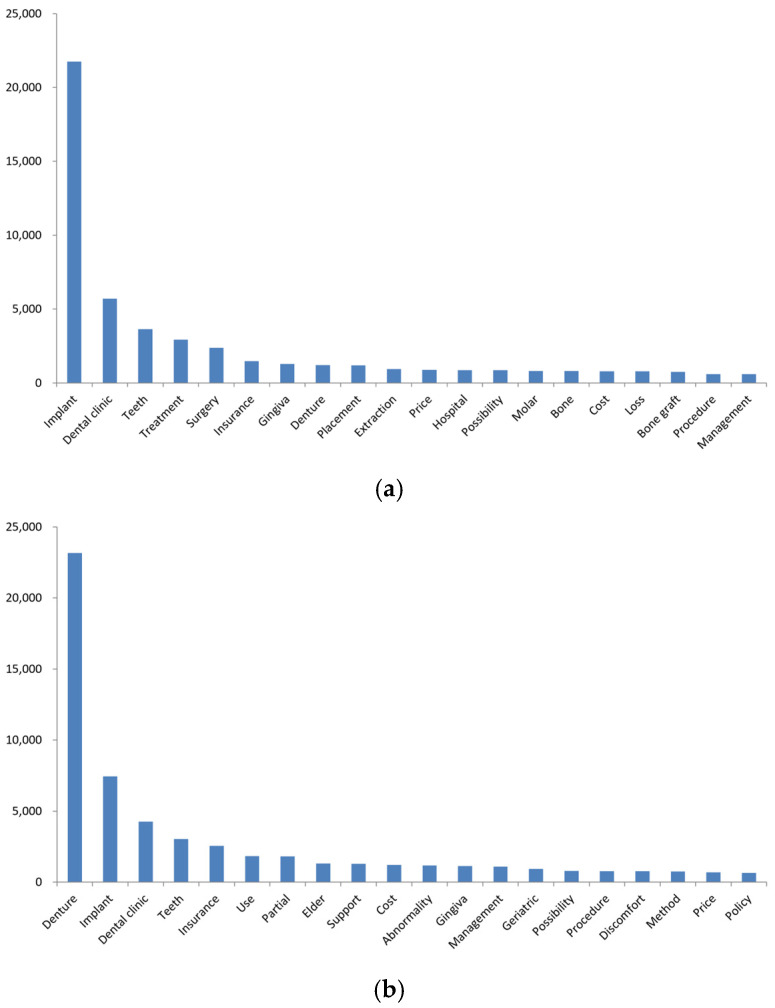
Bar charts for the frequency of the top 20 words: (**a**) dental implants; (**b**) dentures.

Figure 3 and Figure 4 present the word clouds and corresponding bar charts illustrating the frequencies of the top 20 keywords for dental implants and dentures, respectively. Regarding dental implants, “dental clinic,” “treatment,” “surgery,” and “insurance” emerged as the most prominent related keywords. Conversely, within the denture dataset, the term “implant” appeared with notable frequency, while age-related terms, along with “abnormality” and “discomfort,” also ranked among the top search queries.

### 3.2. TF-IDF Analysis

**Figure 5:**
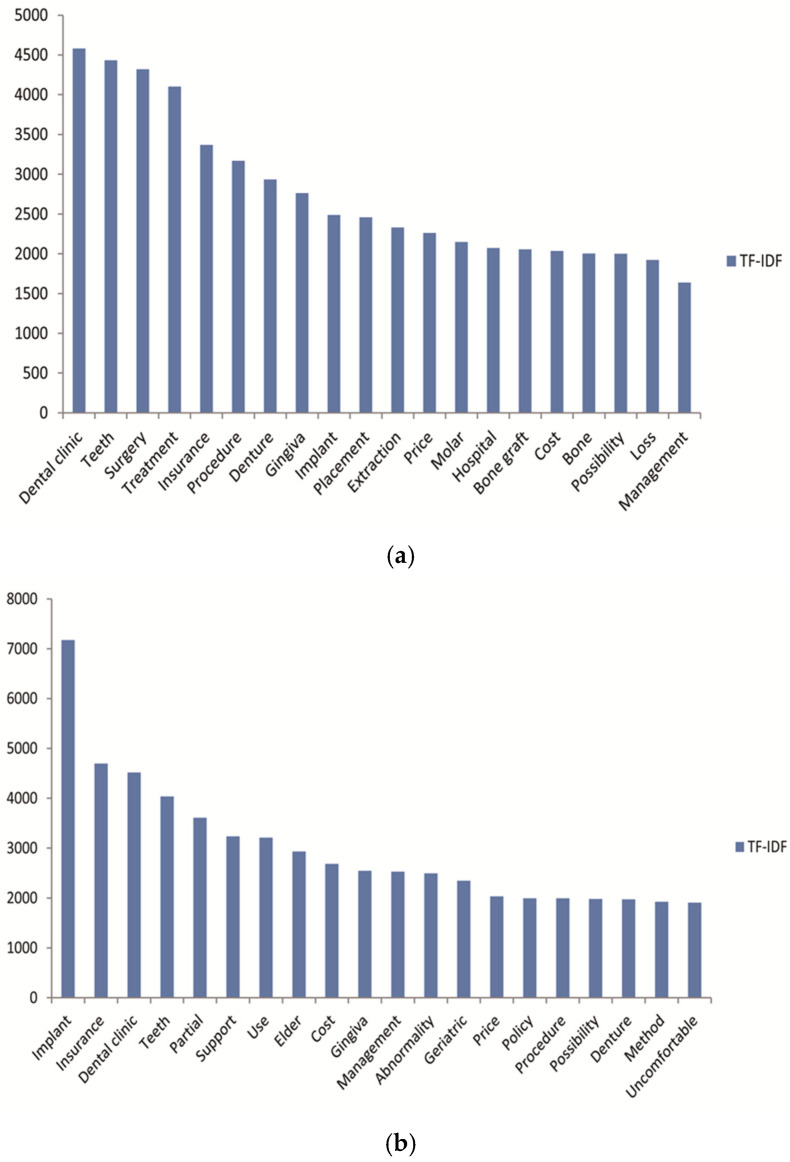
TF-IDF analysis charts: (**a**) dental implants; (**b**) dentures.

Unlike simple frequency counts, TF-IDF evaluates the relative importance and distinctiveness of a word within a specific document across the entire corpus. In the TF-IDF analysis for dental implants, the terms “surgery” and “procedure” achieved higher rankings compared to their positions in the simple frequency analysis (Figure 5a). Conversely, for dentures, the term “insurance” demonstrated a notably high TF-IDF ranking, underscoring its specific relevance to this treatment modality (Figure 5b).

### 3.3. Matrix and Semantic Network Analysis

**Figure 6:**
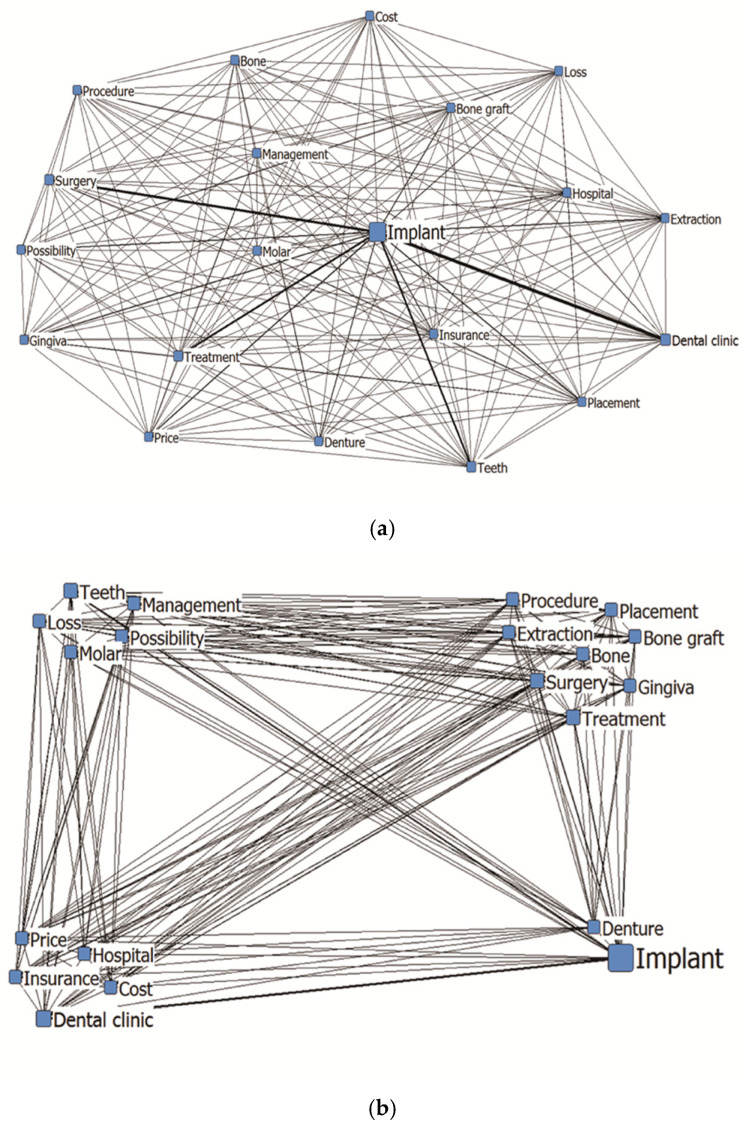

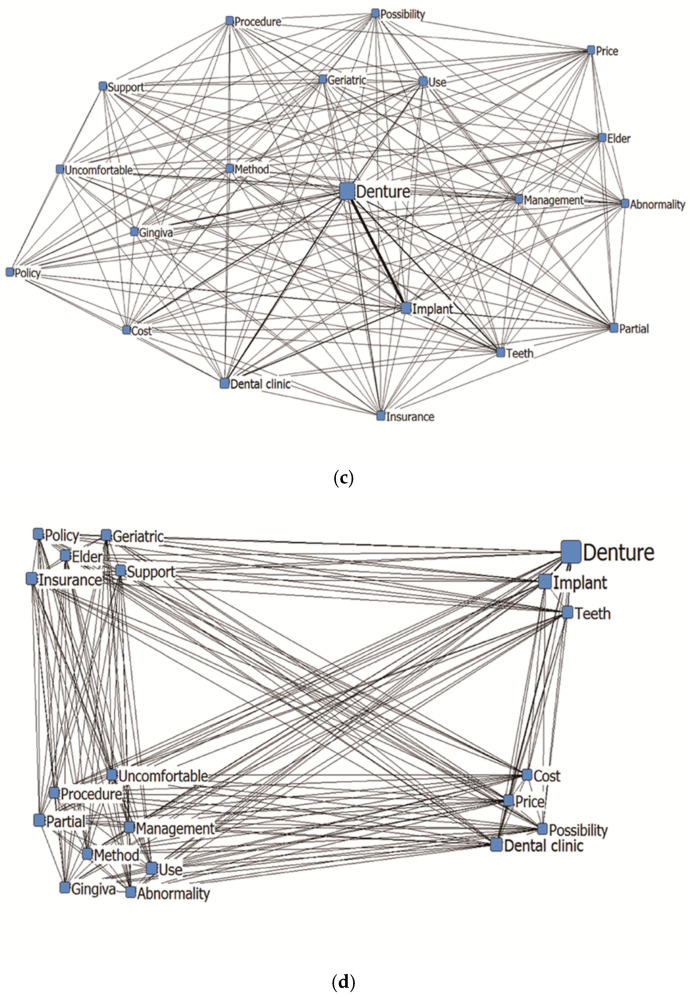
Visualized matrices for the top 20 words weighted by frequency and matrix divided into four clusters based on similarity according to CONCOR analysis: (**a**) Visualized matrix for implants; (**b**) CONCOR analysis for implants; (**c**) visualized matrix for dentures; (**d**) CONCOR analysis for dentures.

To conduct the semantic network analysis, single-mode co-occurrence matrices were generated using the top 20 most frequent keywords identified in the preceding frequency analysis for both dental implants and dentures. The resulting network visualizations for these matrices are presented in Figure 6.

To extract meaningful structural insights from the network, a convergence of iterated correlations (CONCOR) analysis was performed. Because the CONCOR method clusters nodes based on their structural equivalence and correlation similarities, it facilitates the identification of distinct thematic characteristics within each subgroup. The analysis revealed that the keyword networks for both dental implants and dentures were each partitioned into four distinct clusters (Table 1).

### 3.4. Sentiment Analysis

**Figure 7:**
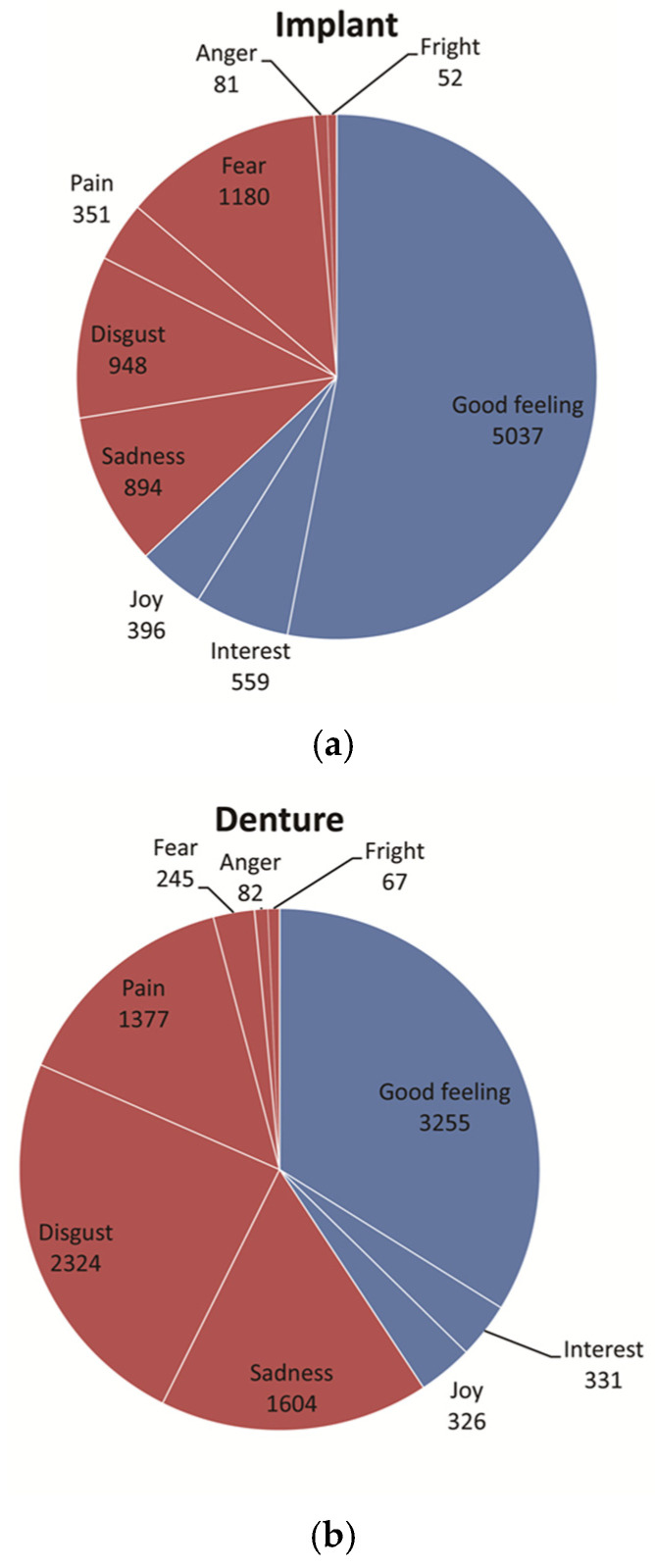
Diagram of sentiment analysis results: (**a**) dental implants; (**b**) dentures.

The sentiment analysis revealed a clear divergence in public emotional responses toward the two treatment modalities. For dental implants, public sentiment was predominantly positive, with positive keywords comprising 63.09% of the total, compared to 36.91% for negative keywords (Figure 7a). Within the positive spectrum, “good feeling” emerged as the most prevalent emotion (53.03%). Conversely, the primary negative sentiments associated with implants were “fear” (12.42%), “disgust” (9.98%), and “sadness” (9.41%).

In stark contrast, overall sentiment toward dentures was largely negative, accounting for 59.30% of the extracted keywords, while positive sentiments constituted only 40.70% (Figure 7b). Although “good feeling” remained the most frequent positive sentiment (33.87%), the negative spectrum was heavily dominated by “disgust” (24.18%), “sadness” (16.69%), and “pain” (14.33%).

## 4. Discussion

This study utilized text-mining techniques to analyze large-scale online data to comparatively evaluate public perceptions of dental implants and dentures. The results demonstrated significant differences in related search terms and public sentiments between the two treatment modalities. Consequently, the null hypothesis of this study was rejected.

The digital revolution has profoundly impacted dentistry, leading to the exponential production of computer-generated data across various dental disciplines [18,19]. Consequently, contemporary dental research is increasingly focused on harnessing the vast potential of digital data to benefit both clinical practice and academic investigation. In recent years, the explosive growth of accessible digital textual data has generated novel insights and expanded research horizons. Within this rapidly evolving landscape of big data analytics, text mining has garnered significant attention. Driven by the rising prominence of artificial intelligence on digital platforms, the application of parallel processing, deep learning, and pattern recognition to textual information has become indispensable. Across diverse sectors—ranging from business models and market research to strategic decision-making—text-mining techniques are increasingly utilized to gain a competitive edge and understand consumer behavior [20].

Building upon our previous research, which confirmed the utility of big data in tracking the growing public interest in dental implants [11], the current study expanded its scope to perform a detailed, comparative keyword analysis of both implants and dentures. Our frequency analysis revealed that “dental clinic” ranked highest for both treatments. This prominence likely reflects the public’s active search for clinical recommendations, compounded by the widespread dissemination of commercial advertising. Because implants and dentures represent significant financial investments that place a substantial economic burden on patients [21], prospective patients exercise caution when selecting a dental provider, frequently relying on SNS and internet searches for medical information and peer reviews [22,23]. For similar economic reasons, terms such as “insurance,” “price,” and “cost” were also searched at high frequencies.

Notably, implant-related searches were dominated by clinical and surgical terminology, including “surgery,” “extraction,” and “bone graft.” In contrast, denture-related queries frequently highlighted “abnormality” and “discomfort,” which likely reflects the inherent functional limitations and physical dissatisfaction commonly experienced by patients wearing removable prostheses, particularly complete dentures [24].

The TF-IDF analysis further reinforced these thematic differences. For implants, “surgery” and “procedure” achieved higher rankings than in the simple frequency analysis, indicating that the surgical aspect is a primary public concern and a defining characteristic of implant-related online discourse. Similarly, the CONCOR analysis revealed that the cluster encompassing the implant surgical process was the most extensive. This aligns with actual clinical practice, where surgical technique is a critical determinant of the success rate [25,26]. Conversely, for dentures, “implant” and “insurance” emerged as top keywords. The frequent co-occurrence of these terms is likely driven by the growing public awareness and adoption of implant-supported or implant-retained overdentures, causing both modalities to be frequently mentioned within the same text passages [27,28].

Although prosthetic material selection critically influences long-term durability, material-specific keywords did not emerge among the top-ranked online search terms in this study. Instead, public discourse was heavily dominated by immediate economic and psychological factors, such as cost, surgery, and pain. This suggests that while patients actively research financial and procedural aspects online, detailed technical decisions regarding specific dental materials are largely deferred to direct, in-person consultations with dental professionals.

Sentiment analysis revealed a significantly more positive public perception of implants compared to dentures, aligning with general clinical consensus that fixed or removable implant restorations yield higher patient satisfaction. Negative sentiments toward implants were primarily associated with the anticipated “fear” of surgical incisions and concerns regarding potential postoperative “pain.” In contrast, negative sentiments toward dentures were heavily characterized by expressions of “disgust,” “sadness,” and “pain.” It is important to note that these online sentiments reflect broad public discourse and perceived anxieties, which may not perfectly correspond to actual individual clinical experiences. Nevertheless, these findings underscore the critical need for clinicians to provide tailored patient counseling and comprehensive preoperative explanations that specifically address the distinct psychological barriers and emotional concerns associated with each treatment modality.

The clinical relevance of this text-mining study lies in its objective identification of public perceptions through unfiltered digital data, which are often difficult to accurately capture in conventional clinical settings. By recognizing these established baseline concerns—such as the distinct association of implants with surgical anxiety and dentures with functional discomfort—clinicians can develop targeted patient education materials and refine informed consent protocols. Ultimately, integrating these data-driven insights can bridge the gap between patient expectations and clinical reality, thereby enhancing the efficiency and quality of patient-provider communication.

In the future, the text mining and natural language processing technologies utilized in this study will serve as foundational tools that can be extended to advanced AI-based predictive models and digital patient profiling in the field of prosthodontics. By expanding these text-driven analytics to clinical data, future AI systems could objectively predict shifting patient expectations and map individual attitudinal profiles prior to treatment. Integrating these advanced data science technologies—while carefully balancing their technological promise and clinical prudence [29]—will enable dental professionals to formulate highly customized, predictive patient communication and education strategies.

This study has several limitations. First, despite applying rigorous filtering criteria, the dataset inevitably included commercial advertisements [30,31]. Specifically, the prevalence of these promotional posts likely inflated the distribution of institution-related and economic keywords—such as “dental clinic” and “price”—thereby potentially overrepresenting the commercial aspects of these treatments compared to purely organic patient inquiries. Second, categorizing sentiments into a binary framework (positive/negative) is inherently oversimplified and may fail to capture the complex, ambivalent emotions of dental patients. Third, analyzing exclusively Korean-language terms from South Korean platforms limits global generalizability, as the findings strongly reflect region-specific socio-cultural and healthcare contexts (e.g., national insurance policies). Finally, online data collection inherently risks selection bias by excluding older populations lacking digital literacy. Future studies should employ multi-dimensional sentiment models and cross-lingual international comparisons.

## 5. Conclusions

This comprehensive big data analysis of widespread online platforms revealed distinct differences in public perceptions between dental implants and dentures. TF-IDF analysis highlighted “surgery” and “procedure” as the most defining keywords for implants, whereas “implant” and “insurance” were central to denture-related discourse. Furthermore, sentiment analysis demonstrated a predominantly positive public attitude toward implants, in stark contrast to the largely negative sentiments and discomfort associated with dentures.

## Figures and Tables

**Table 1:** Four cluster groups according to CONCOR analysis.

	Cluster	Node
Implants	1	Implant, Denture
	2	Procedure, Placement, Bone graft, Extraction, Bone, Surgery, Gingiva, Treatment
	3	Teeth, Management, Loss, Possibility, Molar
	4	Price, Hospital, Insurance, Cost, Dental clinic
Dentures	1	Denture, Implant, Teeth
	2	Cost, Price, Possibility, Dental clinic
	3	Policy, Elder, Geriatric, Support, Insurance
	4	Discomfort, Procedure, Partial, Management, Method, Use, Gingiva, Abnormality

## Data Availability

The data presented in this study are available on request from the corresponding author. The raw data were extracted from publicly accessible web platforms (search engines, social network services, and YouTube), but the compiled and analyzed datasets are not publicly available due to the large volume of text and platform-specific terms of service.

## Abbreviations

The following abbreviations are used in this manuscript:

TF-IDF	Term frequency—inverse document frequency
CONCOR	Convergence of iterated correlations
SNS	social networking services
NLP	natural language processing
